# Editorial: Harnessing Useful Rhizosphere Microorganisms for Pathogen and Pest Biocontrol

**DOI:** 10.3389/fmicb.2016.01620

**Published:** 2016-10-19

**Authors:** Aurelio Ciancio, Corné M. J. Pieterse, Jesús Mercado-Blanco

**Affiliations:** ^1^Dipartimento di Scienze Agroalimentari, Istituto per la Protezione Sostenibile delle Piante, Consiglio Nazionale delle RicercheBari, Italy; ^2^Department of Biology, Faculty of Science, Institute of Environmental Biology, Utrecht UniversityUtrecht, Netherlands; ^3^Instituto Agricultura Sostenible, Agencia Estatal Consejo Superior de Investigaciones CientíficasCórdoba, Spain

**Keywords:** biocontrol, induced resistance, plant growth promotion, rhizosphere microbiology, plant microbe interaction, soil microbiology

Growing demographic trends require sustainable technologies to improve quality and yield of future food productions. However, there is uncertainty about plant protection strategies in many agro-ecosystems. Pests, diseases, and weeds are overwhelmingly controlled by chemicals which pose health risks and cause other undesirable effects. Therefore, an increasing concern on control measures emerged in recent years. Many chemicals became questioned with regard to their sustainability and are (or will be) banned. Alternative management tools are studied, relying on biological, and low impact solutions.

This Research Topic concerns microbial biocontrol agents, root-associated microbiomes, and rhizosphere networks. Understanding how they interact or respond to (a) biotic environmental cues is instrumental for an effective and sustainable impact. The rhizosphere is in this regard a fundamental object of study, because of its role in plant productivity.

## Microbial volatiles and other compounds

Roots are surrounded by a flow of molecules released by microorganisms. Some volatiles may affect growth through different mechanisms, such as biochemical signals eliciting local defense reactions or systemic resistance (Kai et al., 2007; Chung et al., 2016). Yi et al. studied the effects of 2,3-butanediol produced by a *Bacillus subtilis* isolate overexpressing the bud operon synthesis encoding genes. They showed that the isolate persisted on pepper roots more than the wild type, thereby interfering with colonization by fungi. Exudates from isolate pre-treated roots inhibited colonization by *Trichoderma* sp., by another *B. subtilis* strain, and by the soil-borne pathogen *Ralstonia solanacearum*. Application of 2,3-butanediol to roots followed by *R. solanacearum* exposure enhanced the expression of pathogenesis-related (PR) genes. The volatile triggered the secretion of root exudates modulating fitness of soil fungi and bacteria, thus acting as a plant defense inducer.

Bacteria metabolism affects nutrient assimilation. Aziz et al. reported a new mechanism in the plant growth-promoting (PGP) *Bacillus amyloliquefaciens* GB03, which activates genes involved in sulfur assimilation and uptake by *Arabidopsis*. Other transcripts encoding for proteins involved in the biosynthesis of sulfur-rich aliphatic and indolic glucosinolates were also expressed. The enhanced sulfur assimilation increased plant glucosinolate biosynthesis, and conferred protection against the beet armyworm *Spodoptera exigua*.

Bacterial metabolites may also protect plants by inhibiting herbivores (Mithöfer and Boland, 2012). Ganassi et al. reported that also fungal metabolites may have this effect, as shown by a *Trichoderma citrinoviride* isolate, interfering with feeding of the cereal aphid *Rhopalosiphum padi*. Different long-chain primary alcohols (LCOHs) showed a phagodeterrent effect restraining aphids from settling on treated leaves. The LCOHs, perceived through taste receptor neurons and effective at low concentrations, hold potential for insect control in synergy with other compounds.

*Lysobacter* spp. may affect soil-borne pathogens through extracellular enzymes and other metabolites (Folman et al., 2003). Members of this genus appeared abundant in soil suppressive to the root pathogen *Rhizoctonia solani*. Some strains showed *in vitro* activity against *R. solani* and other phytopathogens such as *Pythium ultimum, Aspergillus niger, Fusarium oxysporum*, and *Xanthomonas campestris*. In soil, however, suppression of *R. solani* damping-off on sugar beet and cauliflower was low, and no PGP effect was found on sugar beet, cauliflower, onion and *Arabidopsis thaliana*, likely due to poor rhizosphere colonization. Antagonistic *Lysobacter* spp. are an important source of new enzymes and antimicrobial compounds, although their role in disease suppressiveness needs to be confirmed (Gómez Expósito et al.).

## Soil amendments

The characterization of organic matter amendments enhancing biocontrol and influencing resident soil communities is a growing research field (Bailey and Lazarovits, 2003; Bonilla et al., 2012). Debode et al. studied the effect of chitin on lettuce growth and survival of pathogenic *Escherichia coli* O157:H7 and *Salmonella enterica* colonizing leaves. Chitin addition increased yields and reduced phyllosphere survival of both bacteria, increasing fungal and bacterial biomass in the rhizosphere. An increase was observed for bacterial genera *Cellvibrio, Pedobacter, Dyadobacter*, and *Streptomyces* and the fungi *Lecanicillium* and *Mortierella*. These taxa include species involved in biocontrol, PGP, nitrogen cycle, and chitin degradation, showing a potential for chitin-based amendments.

A positive effect of amendments on soil microbial communities was reported by Vida et al. Composted almond shells elicited suppression of *Rosellinia necatrix*, causal agent of white root rot on avocado, increasing Proteobacteria and Ascomycota, and reducing Acidobacteria and Mortierellales. Beneficial *Pseudomonas, Burkholderia* spp., or Actinobacteria were found only in the amended soil.

## Biocontrol and suppression

Soil suppressiveness is an unfrequent condition, regulating noxious organisms, including root-knot nematodes (*Meloidogyne* spp.; Bent et al., 2008). In two organic horticulture greenhouses in Spain, nematodes decreased progressively in a crop rotation, a decline associated to eggs parasitism by the hyphomycete *Pochonia chlamydosporia*. Controlled assays confirmed suppressiveness, as indicated by lower egg densities and reduced *Meloidogyne* reproduction, observed in non-sterilized soil. A higher microbial diversity was also reported from suppressive soils (Giné et al.).

Entomopathogenic nematodes rely on phoretically associated, insect-killing bacteria (Lewis et al., 2015). In Florida, *Steinernema* sp. and *S. diaprepesi* occupy habitats with different soil properties and water potential. They have opposite migration behaviors in relation to soil water potential, the latter being attracted toward drier soil. After three migration cycles through soil to infect insect larvae, *S. diaprepesi* dominated the nematode community when soil was maintained at 6% moisture, whereas *Steinernema* sp. was dominant at 18% moisture. The nematode responses to water potential and osmotic gradients explained their geospatial patterns. *Steinernema* sp. was recovered only from shallow water tables and moist soils, whereas *S*. *diaprepesi* inhabited ecoregions with well-drained soils and deeper water tables. Differential expression of proteins involved in thermo or mechano-sensation and movement was associated to soil moisture. Proteins involved in metabolism, lectin detoxification, gene regulation, and cell division also differed between the two conditions. Modifying soil moisture in orchards may favor effective entomopathogens, useful in different environments (El-Borai et al.).

Late blight caused by the oomycete *Phytophthora infestans* is the most severe disease of potato (Nowicki et al., 2012). To find alternative solutions to copper-based products, three *Pseudomonas* isolates originating from potato phyllo—and rhizosphere were studied for their protective effect against late blight. When sprayed on potato leaves they survived 15 days in the greenhouse and 8 days in the field. *Pseudomonas chlororaphis* R47 was the most active *in vitro*, and the best protectant in the greenhouse. Its beneficial effects against *P. infestans*, high rates of phyllosphere survival and rhizosphere colonization suggested a potential for application as tuber treatment or leaf spray (Guyer et al.).

*Pseudomonas aeruginosa* is a model system due to endophytism and antagonism toward plant pathogens and pests. Nevertheless, it is also a human pathogen, a trait compromising its exploitation in agriculture (Deredjian et al., 2014). Thomas and Sekhar studied *P. aeruginosa* applications to control other phytopathogenic bacteria. Strain GNS.13.2a isolated from banana rhizosphere significantly reduced the density of native bacteria soon after inoculation. However, its density declined within a week, while a resilient response was observed for native soil bacteria. Assays under axenic conditions or with soil microbiota showed antagonism by the native microbial community, with varying interactive or antagonistic effects.

The use of bacteria, fungi and viruses for weed biocontrol received attention due to prevalence of herbicide-resistant weeds and pesticide bans. Benefits include low environmental impact, higher specificity, reduced costs, and identification of new herbicidal mechanisms. Harding and Raizada reviewed fungal bioherbicides from North America, based on genera *Colletotrichum, Phoma*, and *Sclerotinia*. Bioherbicides also include bacteria of genera *Xanthomonas* and *Pseudomonas* and some viruses. Weed suppression in field conditions is a challenge, as bioherbicides with cytotoxic mechanisms are more sensitive to environmental variables than conventional herbicides.

Bacterial formulations are increasingly produced for sustainable agriculture using biocontrol and PGP species, including endospore-forming *Bacillus* spp. as alternatives to pesticides. Wu et al. reviewed the use of *B. amyloliquefaciens* type strain FZB42, as an application example.

## Mycorrhizae, endophytes, symbioses

Arbuscular Mycorrhizal Fungi (AMF) are primary soil components whose impairment affects the rhizosphere functioning. In their review, Berruti et al. highlighted AMF as valid alternatives to conventional fertilization in sustainable agriculture. Although soil inoculation appears a successful strategy, results may vary, depending on host plant, and fungi. Factors affecting AMF success and persistence include species compatibility with the soil environment, spatial competition and inoculation timing. Genomic and transcriptomic data advanced the knowledge on AMF interactions with the host-plant and other soil organisms, unraveling important factors. AMF can protect host plants against biotic stresses like plant-parasitic nematodes. Mechanisms include enhanced root tolerance, direct competition for nutrients and space, induced systemic resistance, and altered rhizosphere interactions. In a second AMF review, Schouteden et al. emphasized the importance of AMF-based systemic resistance for effective biocontrol of nematode pests.

Besides mycorrhizae, the root niche has been “discovered” also by other endophytic microorganisms contributing to plant health and disease containment (Mercado-Blanco and Lugtenberg, 2014; Hardoim et al., 2015). Bacterial root endophytes control the tomato resistance to *R. solanacearum*. Resistant cultivar Arka Abha showed a bacterial endophyte diversity higher than a susceptible cultivar, including species producing siderophores, HCN and antibiotics, of which three isolates (*Pseudomonas oleovorans, Pantoea ananatis*, and *Enterobacter cloacae*) were effective antagonists. Other resistant cultivars also showed higher prevalence for antagonistic than susceptible bacteria, confirming a role of root-associated endophytes in plant protection (Upreti and Thomas).

Colonization of lettuce roots and rhizosphere by five genetically modified *Streptomyces* spp. was studied by Bonaldi et al. The strains, transformed with a green fluorescent protein marker and apramycin resistance, inhibited *in vitro* the soil-borne pathogen *Sclerotinia sclerotiorum*. In a non-sterile substrate, a transformed strain colonized soil, roots and rhizosphere. When directly inoculated, the bacterium was re-isolated from rhizosphere and roots at densities higher than after seed coating, showing that it is either rhizospheric, and endophytic.

Microbial symbioses also contribute to the competitiveness of invasive plant species. In this regard, the interactions of their microbiome with native species may provide indications for effective biocontrol. A research agenda aiming at developing novel microbial-based biocontrol strategies was proposed by Kowalski et al. based on the invasive plant *Phragmites australis*.

## Virulence and induced resistance

Abscisic acid (ABA) is active in the rice interactions with the blast fungal pathogen *Magnaporthe oryzae* and the antagonistic bacterium *P. chlororaphis* EA105. Spence et al. reported that abscisic acid (ABA) affects plant defense by acting antagonistically on salicylic acid (SA), jasmonic acid (JA), and ethylene signaling. *Magnaporthe oryzae*-produced ABA enhanced plant susceptibility by accelerating pathogenesis through higher rates of spore germination and appressoria formation. Strain EA105 reduced the pathogen virulence by preventing its up-regulation of the ABA biosynthetic gene NCED3 in rice roots and of a β-glucosidase, activating ABA forms. EA105 counteracted the virulence-promoting effects of ABA on *M. oryzae* by inhibiting appressoria formation, preventing spores from increasing ABA biosynthesis and related signaling. ABA implication in plant protection by EA105 involved both direct mechanisms and plant signaling. The role of endogenous fungal ABA was confirmed through the inability of a knoc-kout mutant impaired in ABA synthesis to form lesions on rice, dramatically reducing fungal virulence. Studying the effect of virulence inducers (pH, temperature and acetosyringone concentration) on three homologous genes of *Agrobacterium tumefaciens* (VBP1, VBP2, and VBP3, involved in the T-DNA transfer), Yang et al. showed that vbp2 was affected by pH and by the deletion of vbp1. Results indicated that, in addition to T-complex recruitment, the three homologous genes were involved also in other biological processes.

Martínez-Hidalgo et al. reported how *Micromonospora* strains control fungal pathogens by stimulating plant immunity. This Gram-positive inhabits nitrogen fixing nodules of healthy leguminous plants, with PGP effects. Inoculation of tomato roots with antifungal *Micromonospora* isolates reduced leaf infection by *Botrytis cinerea*, with a durable induced systemic resistance. Gene expression analyses showed that *Micromonospora* stimulates plant defense, enhancing jasmonate-regulated defense pathways, an effects confirmed using defense-impaired tomato mutants. Nodule-isolated *Micromonospora* strains appear as excellent biocontrol agents, combining antifungal activity with the ability to elicit plant immunity.

Pérez et al. showed that the *Trichoderma parareesei* gene Tparo7 encodes a chorismate mutase (CM), an intermediate of aromatic amino acids, essential in protein synthesis and precursor of many secondary metabolites. Decreased levels of Tparo7 in silenced transformants showed reduced CM activity, lower growth rates and mycoparasite behavior against phytopathogenic fungi (*R. solani, F. oxysporum*, and *B. cinerea*) in dual cultures. Higher amounts of aromatic metabolites (tyrosol, 2-phenylethanol and SA) were produced from the silenced transformant, which inhibited growth of *F. oxysporum* and *B. cinerea*. The silenced transformants also showed reduced colonization of tomato root *in vitro*. In greenhouse assays the plants colonized by the silenced transformants were reduced, with a higher susceptibility to *B. cinerea*. The treated plants became yellowish and were defective in JA- and ethylene-regulated signaling pathways, as shown by expression analysis of of lipoxygenase 1, ethylene-insensitive protein 2, and PR protein 1 genes.

Greenhouse trials with fermentation broth of *B. amyloliquefaciens* LJ02 showed effective reduction of cucurbits powdery mildew in China. Li et al. reported that treated seedlings produced superoxide dismutase, peroxidase, polyphenol oxidase, and phenylalanine ammonia lyase at levels higher than control. Free SA, with the PR-1 gene product, increased after leaf treatments, suggesting SA-mediated defense. Secretions from treated cucumber leaves also inhibited fungal spores germination in the rhizosphere. The bacterium and its fermented products showed SA induction, available for powdery mildew biocontrol through systemic resistance.

## -Omics

Recent “-omics” research advances coupled to progressive cost reduction allow a better understanding on many trophic interactions in rhizosphere systems (Massart et al., 2015). The genomes of 12 *Bacillus subtilis* strains with PGP activity were sequenced and analyzed by Hossain et al. The strains exhibited high genomic diversity, except highly conserved *B. amyloliquefaciens* strains (*B. subtilis* group), with 32–90% gene family similarity among *Bacillus* genomes and 2839 core genome genes, similar to *B. amyloliquefaciens* subsp. *plantarum*. Comparative analyses identified genes linked to biocontrol and roots/leaf colonization, including 73 genes from subsp. *plantarum* with functions related to signaling, transport, secondary metabolites and carbon utilization. They encoded several secondary metabolites, with conserved polyketide biosynthetic clusters encoding difficidin and macrolactin. Deletion of secondary metabolite genes in *B. amyloliquefaciens* subsp. *plantarum* showed that expression of difficidin is critical to reduce damage by *Xanthomonas axonopodis* pv. *vesicatoria* on tomato.

Van der Voort et al. sequenced and analyzed the genome of *Pseudomonas* sp. SH-C52. This bacterium, from a *R. solani* suppressive soil, has antifungal activity attributed to the chlorinated 9-amino-acid lipopeptide thanamycin. Its 6.3 Mb genome showed 5579 predicted ORFs, matching *Pseudomonas corrugata* as closest taxon. In silico, secondary metabolite analysis showed six non-ribosomal peptide synthetase gene clusters, including clusters for thanamycin and the 2-amino acid antibacterial lipopeptide brabantamide. Thanamycin is an antifungal whereas brabantamide has anti-oomycete activity, affecting phospholipases of *Phytophthora infestans*. A third lipopeptide, thanapeptin, was discovered, with structural variants active against *P. infestans*. Remaining clusters encode for another unknown lipopeptide. Results indicated a potential for SH-C52 lipopeptides with different antimicrobial activities.

Lichen microbioms were studied by Cernava et al. to analyze antagonism against bacteria and fungi. The *Lobaria pulmonaria* antagonistic community was dominated by *Stenotrophomonas, Pseudomonas*, and *Burkholderia*. 24.5% of isolates were antagonists, accounting for 7% of the metagenome, indicative of an overrepresentation in the culturable fraction. *Stenotrophomonas* bioactive components like spermidine showed PGP effects. Lichens represent important reservoirs for antagonistic bacteria, useful for biotechnological applications.

## Outlook and future challenges

Articles in this Research Topic provide a polyhedral perspective on many issues in which beneficial microorganisms are involved. Data indeed demonstrate that they represent an as yet poorly-explored resource, whose exploitation may actively sustain plant protection and crop production. Given the huge number of microbial species present on the planet, the microorganisms studied represent just the tip of an iceberg. Data produced are, however, informative enough about their genetic and functional biodiversity, as well as about the ecosystem services they provide to underpin crop production. Challenges for future research work concern not only the biology of these species, but also the practices required to protect their biodiversity and to extend their application in the wide range of agricultural soils and systems present in the world. Agriculture cannot remain successfully and sustainable unless plant germplasm and useful microbial species are integrated, a goal for which new knowledge and information-based approaches are urgently needed.

## Author contributions

AC wrote the paper, JM and CP have made direct contributions to the work, and approved it for publication.

### Conflict of interest statement

The authors declare that the research was conducted in the absence of any commercial or financial relationships that could be construed as a potential conflict of interest.
